# Strength in Numbers: Leveraging Mentorship Teams to Support Medical Student Research in Turbulent Research Environments

**DOI:** 10.1007/s40670-025-02575-6

**Published:** 2025-12-12

**Authors:** Stephanie N. Moore-Lotridge, Gloria M. Conover, Luke R. Finck, Jonathan G. Schoenecker, Patrick J. Hu, Diann S. Eley

**Affiliations:** 1 Department of Orthopedics, Vanderbilt University Medical Center, Nashville, TN USA; 2 School of Medicine, Vanderbilt University Medical Center, Nashville, TN USA; 3 Department of Pathology, Microbiology and Immunology, Vanderbilt University Medical Center, Nashville, TN USA; 4 Department of Pharmacology, Vanderbilt University Medical Center, Nashville, TN USA; 5 Department of Pediatrics, Monroe Carell Jr. Children’s Hospital at Vanderbilt, Nashville, TN USA; 6 Vanderbilt Center for Bone Biology, Vanderbilt University Medical Center, Nashville, TN USA; 7 School of Integrative Biological & Chemical Sciences, The University of Texas Rio Grande Valley, Edinburg, TX USA; 8 Department of Medical Education, Texas A&M University College of Medicine, Bryan, TX USA; 9 Office of Medical Student Research, Health Sciences Education, Vanderbilt University School of Medicine, 312 Light Hall, Nashville, TN 37232-0301 USA; 10 Department of Medicine, Vanderbilt University School of Medicine, Nashville, TN USA; 11 Medical School, The University of Queensland, Brisbane, QLD Australia

**Keywords:** Research, Medical student research, Mentorship, Mentorship teams, Student research, Undergraduate medical eductaion

## Abstract

Amid the ongoing changes across the healthcare and research environment—marked by funding constraints, shifting institutional priorities, and increasing clinical demands—faculty are facing mounting challenges to sustaining meaningful research mentorship. At the same time, medical student interest in research continues to rise, driven by both personal curiosity and career-oriented goals. This monograph explores how team-based mentorship models can help bridge this growing divide, offering a flexible and collaborative approach that distributes the responsibilities of guiding student research across mentors with complementary expertise. Drawing from the experience of seasoned faculty mentors across diverse settings, this monograph provides practical strategies to maintain high-quality mentorship despite time pressures and competing priorities. Topics include honest time assessment, shared project design, aligning student timelines with research feasibility, peer mentoring structures, and effective navigation of institutional support. Rather than viewing mentorship as an added burden, this model reframes it as a shared, strategic investment in the future of academic medicine. By embracing mentorship as a team sport, faculty can cultivate environments that celebrate curiosity and perseverance, equipping medical students with the skills, confidence, and passion to meaningfully engage in research—and to carry that commitment forward into their careers as future physician-scholars.

## Introduction

Meaningful research engagement by medical students is essential to promote the development of critical thinking and problem-solving skills through the application of the scientific method [1, 2]. Student motivation to conduct research may be intrinsic, fueled by their curiosity or desire to seek a future career as a physician-scientist [3, 4]. Likewise, students may be extrinsically motivated, given that research productivity has become an integral element in residency applications, particularly for competitive specialties and programs [5–7]. Aligning with these motivations and a desire to establish a lifelong professional identity of curiosity, many medical schools have developed curriculum elements that enhance student research engagement to create meaningful experiences with tangible outcomes.

A key element to the success of these medical student research curricula is recruitment and engagement with expert faculty mentors. These mentors are responsible for guiding students through the research process, from conceptualization to publication, ensuring that they gain the skills, knowledge, and confidence needed to contribute meaningfully to the field [8]. With ongoing changes across the research and healthcare landscapes, specifically access and availability of research funding, mentors may find themselves juggling additional competing priorities, impacting their capacity to work with students. Given these limitations, this monograph has been curated by seasoned mentors, from a variety of training backgrounds including an international perspective, to provide tips and tricks for faculty to continue to support medical student research in uncertain research environments.

Traditionally, mentorship occurs as a one-on-one relationship, where an individual mentor guides a single mentee or a group of mentees simultaneously, who may be all from the same or a tiered level of training [9–11]. This form of mentorship can be challenging when available time for the mentor is limited and/or mentees do not see an immediate value in nurturing a mentorship relationship. Thus, in this article, we will specifically highlight team mentorship, a newer model, involving a group of mentors, who collectively support one or a small group of mentees, promoting different complementary perspectives and collaborative learning from a variety of disciplines [12, 13].

The goal of this monograph is to guide and support the development and retention of research mentors who are integral in supporting medical student participation in high-quality research projects. While multiple publications have focused on the students’ perspective to improve engagement with meaningful research and enhance productivity [14], to date, less guidance [8] has been published to support the mentors of these students. This is especially important in the current healthcare/research landscape. Over the past 5 years, publications have reflected on barriers experienced by mentors that limit their engagement with students wishing to conduct research. This includes misaligned research expectations, limited time, competing priorities, mentor burnout, and a lack of research infrastructure [2, 15–19]. While the mentor cannot control all elements (e.g. institutional/department culture, government mandates, or research infrastructure), the purpose of this article is to provide actionable tips to empower mentors and mentor teams engaging with medical students as they conduct research in an uncertain research environment.

### Research is a Team Sport—And So is Mentorship

**Fig. 1:**
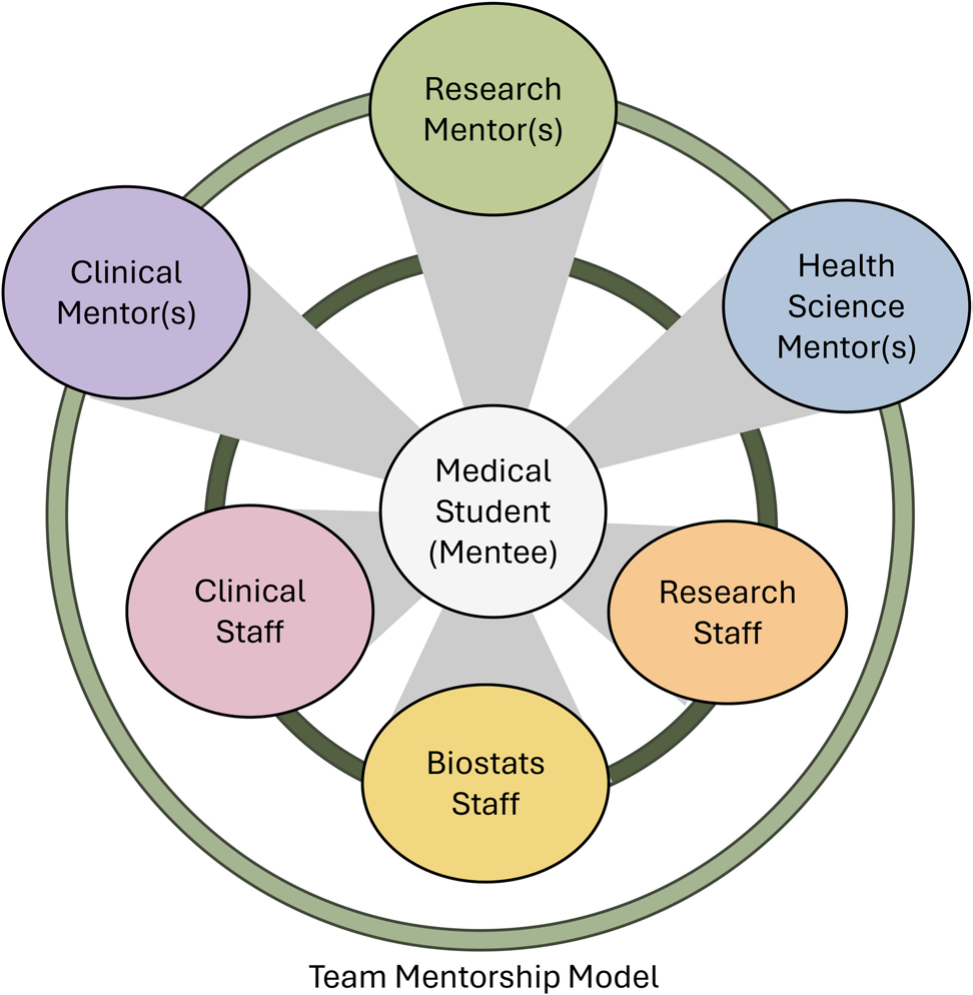
**Team Mentorship Model:** This model involves a group of mentors, with variable expertise, working together to support one or a small group of mentees. Each mentoring individual will contribute different perspectives and skills to the mentee, helping to support holistic and engaged training. For example, research, clinical, and health science mentors will include expert faculty who are responsible for the overall direction of the project, hypothesis generation, data interpretation, and scientific communication. Health science mentor(s) may broadly include pharmacists, geneticists, social workers, epidemiologists, bioinformaticians, etc. Likewise, this team will also include knowledgeable clinical, research, and biostatistics staff, who may contribute to “first line” hands-on training (dark green circle) for research skills and analysis

Prior to recent reductions in US government research funding and the subsequent changes in higher education research support structures, the perceived systemic barriers for research mentors engaging with medical students remained. These primarily include limited time, perceived inexperience, lack of confidence, or aptitude in conducting academic research. As such, we propose that team mentorship, where a medical student is mentored by a group of clinical and/or research professionals with complementary disciplines, with the goal of mitigating some of these barriers (Fig. 1). By including the perspectives of groups of professionals, particularly those with distinct but complementary expertise, mentors can help ensure that the student is adequately supported both in time and skills to meaningfully engage in research. For example, clinicians conducting research in a field may partner with research scientists and statisticians, who together can collaborate to train students in research skills relevant to the project, while also keeping students accountable and providing expert mentorship throughout the completion of a research project. By working together as a mentorship team, faculty support each other’s time investment in medical student research and other competing priorities.

While team mentorship may be a helpful strategy for overcoming time as a barrier to mentorship, this model does come with limitations, such as those highlighted by Mancuso et al [20]. For example, team mentorship works best when goals and research question(s) are aligned to better support study design/goals, timeframes, and feasibility of the work. Furthermore, it is important to ensure clarity of the message from the team of mentors to the student. Therefore, we agree with Mancuso *et. al.* recommendation that the mentorship team designate a leader or spokesperson [20] who will be responsible for communicating action items from the mentorship team, such as changes and timeline adjustments, with the mentee(s). This is important to avoid overwhelming a student who will likely be a novice and eager to impress their mentors. Considering these potential challenges, early focused discussions between the mentorship team are warranted, including detailed discussions of time availability, authorship considerations, and each member’s anticipated contributions to the project to provide a clear path for the student. Finally, continual communication throughout the project is necessary to ensure consistent messaging, actionable feedback and guidance from the mentorship team to the mentee.

### Be Honest with Your Time for Research and Mentorship

As highlighted above, time availability for research mentorship is a well-identified and long-existing barrier to faculty engagement. While this can be mitigated with the help of a mentorship team, the team must be aligned regarding their time available to plan, mentor, and complete deliverables (abstracts, manuscripts, etc.) that likewise align with student deadlines. From our experience, the most effective mentorship teams are those that discuss these elements early and have honest discussions regarding their availability and anticipated contributions before engaging with a mentee. A benefit of a team-based approach is that it can account for the natural ebb and flow of an individual’s availability, allowing others to take on a greater role during these phases of a project, without negatively impacting the mentee(s) training or deadlines. Furthermore, given the current healthcare/research landscape, faculty are juggling multiple priorities, such as increased clinical responsibilities, grant writing expectation, and variable teaching loads. A team-based approach can spread the research and mentorship responsibilities across individuals, alleviating the load on a single faculty member.

### Understand your Mentee’s Timeline

Undergraduate medical students conduct research on a variety of timelines. This can include dedicated summers during the preclinical phase, part-time during the academic years intertwined with other scholastic responsibilities, dedicated time during their post-clerkship phase, or even a dedicated full research year. As a mentor, it is important to understand your mentee’s research schedule as this can influence the feasibility and success of your research study.

When designing a research study, the mentorship team should discuss the mentee’s availability and commitment to the project to set a mutually feasible timeline. This timeline should then be used by the mentorship team to ensure: 1) Approvals and study design are completed to allow research to begin as expeditiously as possible, 2) The proposed study is feasible, regarding the student’s time and knowledge with mentor support, and 3) The anticipated deliverables (presentations, abstracts, manuscripts) can be obtained before necessary deadlines (i.e. graduation, residency application submission, etc.). In situations where a medical student may be involved in a multi-year project, it is imperative for the student and their primary mentor to delineate the project’s short and long-term goals and define the specific role of the student and what they are expected to accomplish. These defined goals should then be communicated by both the student and mentor to the larger team (where appropriate) and adjusted as the project progresses. Finally, we recommend that the students and the mentorship team define a clear meeting schedule in all cases, which may be monthly to bi-annually for long-term projects or more frequently (i.e. weekly) for short-term projects.

### Sound Project Design is the Foundation for Medical Student Research

Project design is critical for the success of any research project, which is further magnified with the inclusion of a mentee [8]. As the research mentorship team establishes the overarching goals of a research project (Table 1), these must be communicated clearly to the mentee(s). Furthermore, aligning with recommendations from MacDougal & Riley, we postulate that inclusion of a mentee during the project design phase, when possible, can enhance a trainees’ critical thinking and problem-solving skills, in addition to fostering stepwise ownership of the proposed work [8]. For example, building upon the foundational discussions with their mentor(s) (Table 1), we ask our mentees to assist with the refinement of the research question (PICOT elements; population, intervention, comparison, outcome, time) and proposed variables, the identification of strengths and weaknesses of the study, the planning of the study design and associated analysis, and anticipated outcomes (Table 2). Importantly, this is done under the guidance of faculty mentors and further ensures that the mentee understands the foundational information, goals, and potential limitations a study may face. To support this process, we recommend using Tables 1 and 2 together to collaboratively design a research project with your mentee.

**Table 1:**
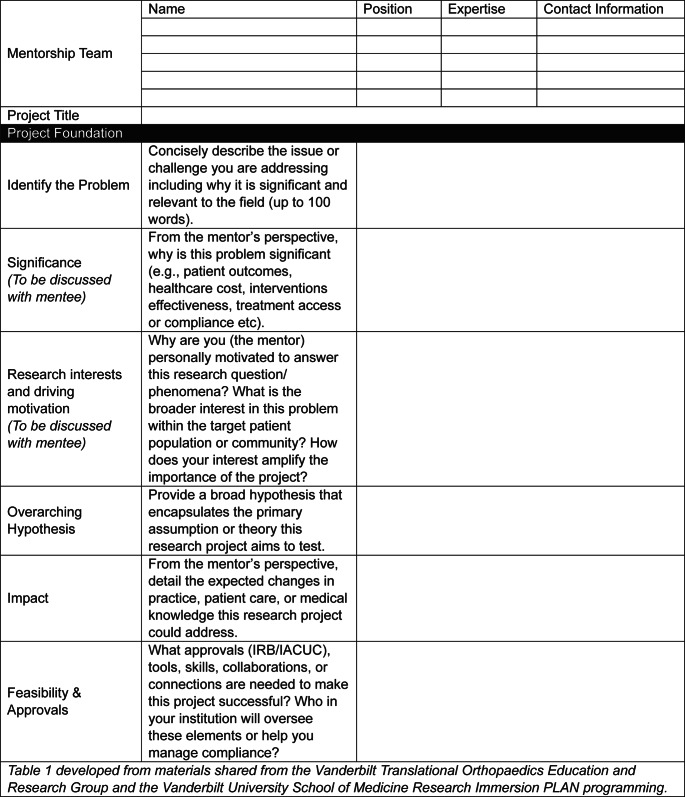
Project Planning Form Completed by Mentor & Mentee Together

**Table 2:**
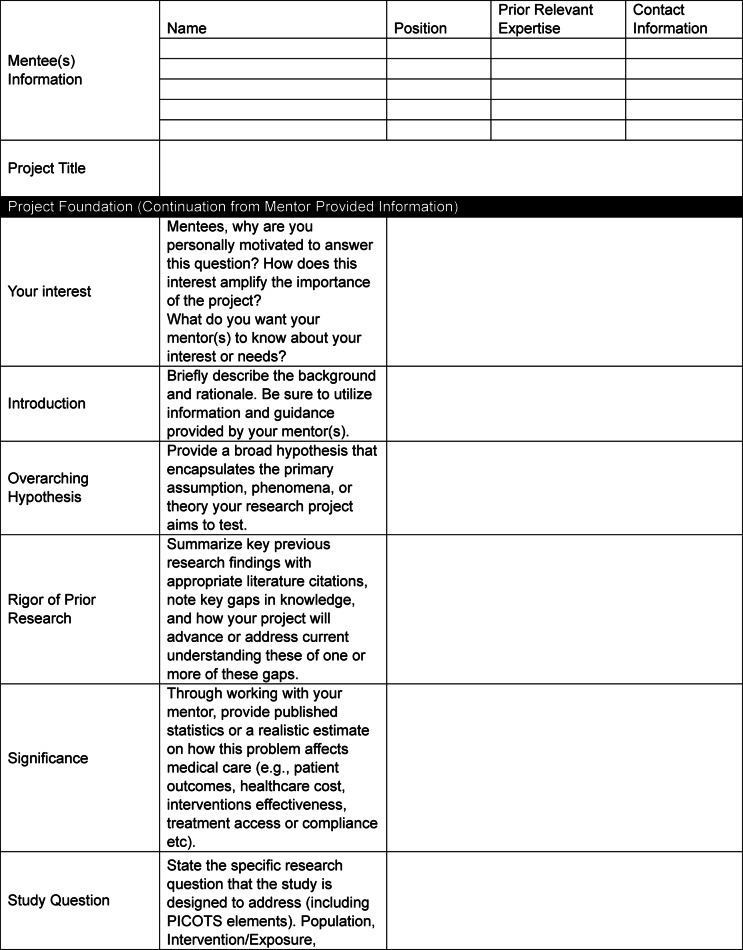

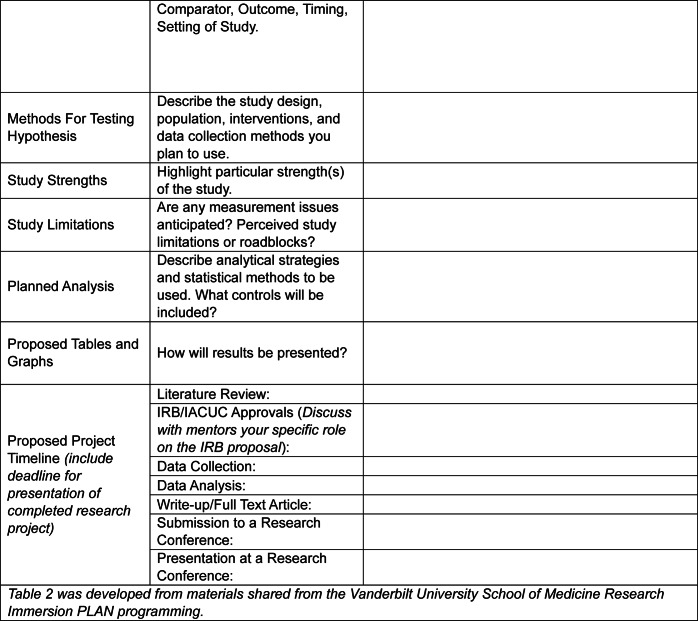
Project Planning Form-To be filled out by Mentee

In cases where mentored research time is limited, such that the mentees would be unable to actively participate in the design of a project, we recommend the inclusion of one or more of the following activities to support the student’s development:

1. Shadow Research Design Discussions: Mentors will meet with mentee(s), present the study they will be contributing to, and share the rationale for the prior study design decisions including known resources and/or barriers/challenges that influenced the decisions. Mentors will encourage mentees to ask questions and propose alternative design elements for future consideration. This structured discussion can provide a valuable learning opportunity by helping trainees understand how methodological choices are made in real-world contexts, fostering critical appraisal skills, and equipping them to thoughtfully contribute to study design in subsequent projects.
2. Comparative Design Appraisal: Students are provided with a published study on a related topic and asked to compare and contrast its design with that of their current project. Under mentor guidance, mentees will analyze the study hypothesis, approach, methodological strengths, limitations, and contextual factors that influenced each approach. This activity can reinforce evidence-based reasoning while directly linking theoretical design principles to the practical realities of their own research experience.
3. Dream PICOT Trial: Mentees will be guided to design their own “ideal” study around a research topic that is different from the work they are conducting. They will be asked to construct their “Dream PICOT” framework following the award of an imaginary $10 million grant. Mentors will facilitate a discussion of the mentees’ proposals, highlighting trade-offs in feasibility, resources, ethics, and timeline. This activity can provide a creative and engaging avenue for trainees to practice research question formulation, appreciate the influence of pragmatic considerations on study design, and develop flexibility in balancing scientific rigor with practical feasibility. Importantly, this activity can either be done in one session or be carried out over several sessions, depending on the timeline and availability of the student and mentors.
4. Tiered Ownership: Students begin with a faculty-driven research project design to ensure feasibility within the limited research timeframe. Within this framework, mentees are invited to identify one area—such as conducting a secondary analysis, refining the research question for a subgroup, or exploring an additional variable—where they can exercise greater autonomy. This activity can provide a tangible opportunity to practice essential elements of study design while maintaining timely project completion, thereby fostering both ownership and skill development in a manageable scope.

### Don’t Forget about Institutional Ethics Approvals

When designing a research project, the mentorship team should consider the necessary study approvals early. This is becoming increasingly more important, as research infrastructure (such as personnel and resources) may have changed, leading to delays in study review and approvals. Likewise, new guidelines and mandates may limit certain topics of research. Within the United States, this includes ethical approvals such as institutional review board (IRB) for human-based studies, institutional animal care and use committee (IACUC) for animal studies, or institutional biosafety committee (IBC) approval for in vitro experimentation (Table 1). Given that the mentors will hold ultimate responsibility for the ethical conduct of the research, as well as institutional accountability, we recommend that the mentors take the lead on obtaining these approvals, initiating submissions ~ 3–6 months before the initiation of the research project. With the changes many institutions are experiencing in research infrastructure, this timeline may need to be longer.

Submitting the IRB or IACUC application ensures that the faculty member fully understands and is accountable for the study’s design, risks, and compliance with regulatory requirements. Compared to mentees, faculty mentors often have greater experience and institutional knowledge to navigate the complexities of IRB/IACUC/IBC institutional guidelines, helping to reduce the risk of errors, omissions, or misinterpretations that could delay approvals further; thereby ultimately making the process timelier.

Importantly, timelines for approval should also be considered relative to the mentee’s timeline. In cases where time with a mentee is limited, mentors may need to initiate submissions even earlier (6–9 months) if preliminary studies, data collection, or data cleaning are necessary prior to a mentee joining the project. Alternatively, if time permits, these applications allow faculty to model best practices in research ethics and compliance to mentees, reinforcing the importance of rigorous ethical standards in research. Thus, faculty members can guide students through the application process, providing educational value while ensuring the submission aligns with institutional and regulatory compliance.

### Conduct a Needs Assessment Prior to Starting Research- What are their goals? What are your goals?

When conducting research with medical students, successful mentorship hinges on understanding the unique needs, goals, and aspirations of the mentee while aligning them with the objectives of the research project and the mentorship team’s priorities. Each student enters research with different experiences, skills, interests, and career ambitions. Reports agree that the intrinsic motivation for novice students to begin research should lie in curiosity and joy [21] and mature to seek to impact and advance their specialty clinical patient care [22]. Additionally, reports suggest it is crucial for mentors to dialogue with their students about the benefits of engaging in formal research [23–25].

By fostering open communication, mentorship teams should identify their student’s motivations and tailor the research experience to provide both skill development and professional growth. At the same time, aligning the student’s efforts with the broader goals of the project ensures mutual benefit: the student gains valuable critical appraisal of scientific research discoveries through collaborative learning opportunities, while the mentorship team moves their research program forward, progresses toward scholarly outputs, and long-term research aims. This alignment not only enhances research productivity but also strengthens the mentor–mentee relationship, fostering a collaborative environment that supports the student’s success while advancing impactful contributions to medical research.

We propose that communication regarding these matters should start early during the developmental phase and be continued throughout the project. This allows mentorship teams to discuss any changing needs of the mentee and respond to roadblocks or challenges associated with the research in real-time; including future challenges that may be unforeseen at present.

### Expectation Setting: What do you expect of your Mentee? What does your mentee expect of you?

Effective mentorship for medical students conducting research requires clear expectation setting from both the mentorship team and mentee [26, 27]. At the outset, the mentorship team should align their expectations for the mentee and clearly articulate these to the student, such as commitment to timelines, proactive communication, intellectual curiosity, and ownership of assigned tasks [28]. Similarly, the mentee should share their expectations of the mentors, which may include guidance on research design, feedback on drafts, and career advice. Given the broad range of mentors that may make up a mentorship team, some individuals may be better suited to assist with career advice while others may guide research design or scientific writing; together this can lighten the responsibilities for any single mentor.

As highlighted above, it is important to note that successful mentorship often extends beyond research supervision—it may also involve serving as a coach and sponsor. Team mentorship can help meet these needs of a student by providing mentors with variable expertise. As a coach, the mentors support the student’s professional and personal development by fostering critical thinking, building resilience, and teaching essential research and clinical skills [14, 29]. This role should address challenges, helping the student manage their workload, navigate their busy academic schedule, and establish life-work balance to prepare them for challenges they will encounter as future physician-scientists or clinicians. As a sponsor, the mentors will actively advocate for the mentee’s advancement, helping them build networks, recommending them for research presentations, reviewing their grant proposals or manuscripts, or connecting them with collaborators [30].

Discussing these roles across the mentorship team with the mentee during meetings early in the relationship ensures that the mentorship teams can meet the mentee’s broader developmental needs while balancing the research project and external competing priorities. By clarifying and integrating these roles, the mentor–mentee relationships can become a holistic partnership that maximizes the student’s growth and success.

### Establish Additional Layers of Support: Use of Peer-Peer Mentorship and Mentee-Teams

While faculty mentorship is a cornerstone of research success, the inclusion of near-peer mentors or mentee teams can markedly enhance the experience and outcomes for students engaging in research [31, 32]. Working alongside peers fosters camaraderie, creating a supportive environment where students can share challenges, celebrate successes, and learn from one another. Peer collaboration often leads to greater achievements, as diverse perspectives and skills combine to tackle complex research questions and execute projects efficiently [33–35]. Additionally, having peers work together on a research project helps accommodate variable clinical or academic schedules and availability among students, ensuring that progress can continue even when individual team members face academic or personal obligations. Collaborative work also increases opportunities for shared accomplishments, such as co-authored publications and joint presentations at conferences, which amplify the professional benefits for all research team members. Beyond academic success, these peer interactions build essential life-long skills in appreciative inquiry, emotional intelligence, teamwork, communication, and leadership, preparing students for the collaborative nature of clinical practice and research in their future careers [36, 37]. By leveraging the power of peer mentorship, medical students can enhance their research experience and establish lasting professional relationships that may lead to long-term collaborations and camaraderie in research and academia [38].

When incorporating peer mentoring into a research team, mentors should ensure clear expectations and fair credit allocation are established to help foster a positive and supportive collaborative environment. It is essential to work with mentees to define their roles and responsibilities while ensuring that everyone can champion their own project(s). For example, collaboratively building a shared retrospective database while allowing each student to use the data to answer distinct research questions can be highly beneficial as it promotes individual ownership and intellectual growth while leveraging the group’s collective effort to achieve greater efficiency and depth in the research. Regular communication and progress checks can help maintain balance, ensure fairness, and reinforce the value of teamwork.

### Scholarly Research Deliverables

Mentors play a pivotal role in guiding students toward producing meaningful and rigorous research deliverables, which are critical to their students’ academic and professional success. Mentorship teams should work with their mentees early to identify appropriate scholarly deliverables for their career stage, such as conference abstracts, poster presentations, or manuscripts sharing their work. Furthermore, mentors and mentees should work together to establish a feasible timeline, including deadlines and planned time for internal review, to facilitate timely project completion. In addition to supporting the student, the use of a mentorship team can likewise support early career faculty who may also need support when navigating scientific writing and academic publishing. Thus, mentors and mentees together can build a research portfolio that showcases their skills, contributes to their field, and enhances their competitiveness for future opportunities. Consideration and coordination with career advisors, Academic Affairs, Student Affairs and Research Deans, to assist learners with aligning their research goals with their academic responsibilities is crucial. Working in concert, these leaders can support both mentors and learners with identifying optimal times to identify projects and mentors during an often-packed curriculum.

### Resources for You and Your Mentee

Resources available to mentors/mentees to support research will vary between and across institutions, especially given ongoing changes across the research and healthcare landscapes. In Table 2, we have outlined some common resources mentors/mentees should consider identifying at their institution that can support their research and dissemination of findings. Briefly, this may include institutional library resources, software, communication tools, data storage and management, or career development resources focused on research skills, academic publishing, networking, or institutional funding.

Furthermore, we recommend that both mentees and mentors look beyond their home institutions for resources and support for their research endeavors. This may include national and international research organizations, clinical organizations, professional societies, collaborative research networks, private foundations, advocacy groups, international collaborators, corporate partnerships and industry sponsors, community and patient engagement resources, data repositories and open data sources, and pharmaceutical or biotechnology companies.

### Navigating Institutional Support

Much like time, money (i.e. institutional support or grants) has long been cited as a major barrier for mentors to engage in research. This has become even more evident in recent times, where mentors may have trouble securing funding due to high competition for federal dollars, due in part to the fewer requests for proposals (RFP) available today that were previously available for NIH or NSF grant funding agencies. Additionally, faculty may experience new limitations when advocating within their institutions for the resources necessary to support their research projects and their mentees’ professional growth. In the face of these limitations, a mentorship team may make negotiations for institutional or departmental support less onerous by leveraging their collective strengths, aligning goals, and presenting a unified case for the value of their research initiatives.

Obtaining funding for research is an ongoing challenge. Beyond federal funding, it is the responsibility of the mentorship team to think creatively and apply for alternative funding opportunities. This may include internal sources such as college bridge funding, research grants, university-level teaching grants, departmental funds, and institutional awards to sponsor mentees. External sources that can be considered include funding from research and clinical organizations, professional societies, collaborative research networks, private foundations, advocacy groups, corporate partnerships and industry sponsors, and pharmaceutical or biotechnology companies.

Furthermore, mentorship teams can leverage institutional resources in their search for funding. This may include programming from research offices, grant-writing support teams, or shared laboratory facilities (e.g. cores) to maximize research efficiency and cost-effectiveness. Additionally, the team mentorship model proposed here could support pathway programs focused on recruiting pre-medical undergraduate students interested in continuing research during their undergraduate and graduate level medical training. Thus, proactively building a case for the value of research—not only for academic advancement but also for enhancing institutional reputation and attracting high-caliber students—can help a mentorship team gain the necessary support to maintain research success in the current turbulent and unpredictable research environment.

### Celebrating the Joy of Discovery and Advancing Medicine

Amid the Spring 2025 sweeping changes in the United States healthcare and medical research landscape, it is easy for both mentors and medical students to feel disheartened and discouraged regarding the future of academic medicine. During these times, mentorship and camaraderie can be a powerful force for hope – helping us to celebrate curiosity, resilience, and the shared mission of advancing science and medicine together. As such, we propose that team science and the use of mentorship teams can be used proactively to help mitigate the loss of research trainees and young faculty by creating partnerships that leverage each other’s research expertise and available resources.

Beyond helping students navigate the larger impact of changes on medical research, mentorship teams remain critical to help frame day-to-day successes and failures in research with their mentees. This can include celebrating their mentees’ successes (e.g. publications, awards, abstracts, ect), their resilience in the face of a failed experiment, or their professional skills when working with a team. By intentionally shaping the environment and perspective around research in medical school, mentorship teams can profoundly influence their mentees’ perception of research and their desire to continue with scholastic engagement in their future careers.

## Conclusion

This monograph offers best practices for mentors guiding medical students in research, emphasizing the benefits of utilizing a team mentorship approach, with mentors from complementary disciplines, to overcome barriers such as limited time and experience. This work reflects on the importance of honest and frequent communication between mentorship teams and mentees about availability, project design, necessary approvals and timelines, aligning mentee goals with research objectives, setting clear expectations, identifying deliverables and timelines, securing institutional resources and support, and celebrating successes while framing failures as growth opportunities, ultimately fostering a supportive environment for meaningful experiences and tangible outcomes.

Ultimately, team mentorship reframes research guidance as a shared investment in the next generation of physician-scholars. Yet, for this model to be successful, mentors must remain vigilant to its challenges, ensuring that coordination, accountability, and realistic expectations are woven into the mentorship fabric alongside curiosity, resilience, and discovery.
